# Blowing Kinetics, Pressure Resistance, Thermal Stability, and Relaxation of the Amorphous Phase of the PET Container in the SBM Process with Hot and Cold Mold. Part II: Statistical Analysis and Interpretation of Tests

**DOI:** 10.3390/polym12081761

**Published:** 2020-08-06

**Authors:** Paweł Wawrzyniak, Waldemar Karaszewski

**Affiliations:** 1 Institute of Machine Design Fundamentals, Faculty of Automotive and Construction Machinery Engineering, Warsaw University of Technology, 82-524 Warsaw, Poland; 2 Department of Machine Design and Motor Vehicles, Faculty of Mechanical Engineering, Gdansk University of Technology, 80-233 Gdańsk, Poland; walkaras@pg.edu.pl

**Keywords:** PET, SBM process with hot mold, microcavity, blow kinetics, relaxation of PET amorphous phase, power of ANOVA test

## Abstract

The technology of filling drinks without preservatives (such as fresh juices, iced tea drinks, and vitaminized drinks) is carried out using hot filling. Mainly due to the production costs and lower carbon footprint, polyethylene terephthalate (PET) bottles are increasingly used in this technology. In this paper, the main aim is to describe and interpret the results of statistical analysis of the influence of the temperature of the blow mold in the SBM (stretch blow molding) process and the method of hot filling on the macroscopic and microscopic bottle properties. The macroscopic bottle properties were defined by the thickness profile, pressure resistance, thermal stability, and the coefficients of blowing kinetics. In addition, the influence of the SBM process on the microscopic PET material properties (in the bottle) relative to the microscopic preform properties was analyzed. The microscopic properties were defined by the degree of crystallite, density, and relaxation of the amorphous phase of the PET material. For this purpose, response surface experiments were performed for the two analyzed factors, i.e., the temperature of the blow mold and the method of hot filling. The sample size was investigated to determine the minimum number of repetitions (number of bottles in the measurement series) required to achieve acceptable measurement uncertainty. The research conducted shows that, despite fulfilling the postulate of acceptable measurement uncertainty, in terms of the power of ANOVA (analysis of variance) in DOE (design of experiment), the accepted number of bottles in the measurement series is too small. The tests of the bottle material density, material crystallite, and relaxation of amorphous phase relative to the preform material density, material crystallite, and relaxation of amorphous phase show that microcavity effects occur during the deformation of the PET material, and that these are associated with the orientation of the microstructure. The blow kinetics study shows that there is a gradient of flow of the bottle material over the thickness of the bottle wall during blowing, and it has been deduced that the air temperature between the blow mold and the wall of the blown bottle has an impact on the kinetics of blowing the bottle.

## 1. Introduction

A broad review of the literature on the SBM process with cold and hot molds is presented in other articles [1,2,3,4]. The first part of the paper contains a literature analysis, the purpose of the research together with a description of the methodology of experimental research and its results [5]. Therefore, the purposes of this article are:

- to present the number of measurement repetitions so that the measurement error is within acceptable limits relative to the actual value of the measured characteristic;
- to present the DOE analysis and power of ANOVA tests;
- to present the blow kinetics in aluminum blow molds—shear phenomena along the wall thickness of the blown bottle have been noticed, and it has been deduced that the air temperature between the blow mold and the wall of the blown bottle has an impact on the kinetics of blowing the bottle; and
- to present the relaxation of the amorphous phase—the occurrence of micro-cavitation phenomena has been deduced in the PET material of the blown bottle.

## 2. ^13^C-NMR Analysis of PET Preform Material

**Figure 1:**
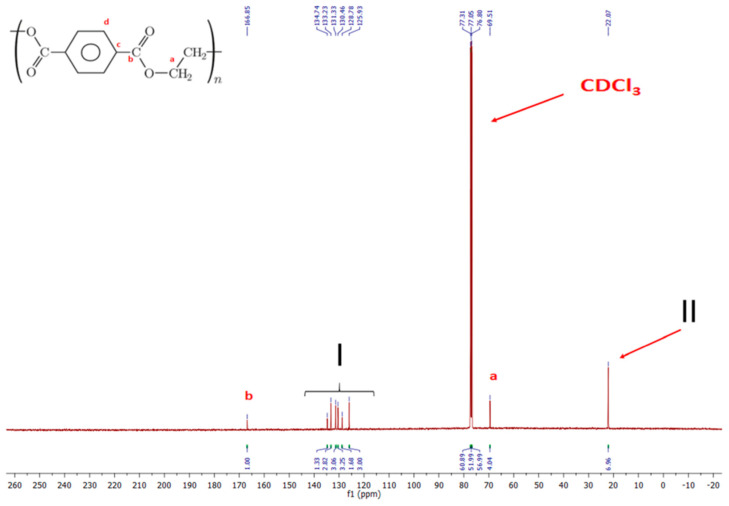
^13^C-NMR spectrum of preform material: I, signals from carbons “c” and “d” and aromatic ring of unknown polymer; II, signal from the structure $Ar-CH_{2^{-}}$ or $Ar-X-CH_{2^{-}}$ (where X is another atom, e.g., S).

Figure 1 shows the ^13^C-NMR (carbon-13 nuclear magnetic resonance) spectrum of the preform material (cut out around Point 4 of the blow molding kinetics marks on the preform [5]), in which signals from carbons “a”, “b”, “c”, and “d” can be distinguished. There should be twice as many carbons “d” as carbons “a”, “b”, and “c”, but this is not the case, and there are peaks, which should not be in pure PET. The ^13^C-NMR spectrum indicates the presence of PET copolymer rather than pure PET. The ^13^C-NMR spectrum was performed on a 500-MHz Brucker spectrometer at 295 K in a solution of deuterated methylene chloride with the addition of hexafluoropropanol. The analysis of the ^13^C-NMR was done at the Polymers Division of The Centre of Molecular and Macromolecular Studies in Lodz.

Deeper analysis of the peaks in Figure 1 indicates the presence of an additional structure that does not have a carbonyl group (because there is only one signal from the carbonyl group with a chemical shift of 166.85 ppm, corresponding to the structure of PET material). However, this structure certainly has an aromatic ring with which the aliphatic carbon (–CH_2_–) is directly connected or the aromatic ring is connected with another atom (e.g., sulfur with the –CH_2_– group). The presence of additional –CH_2_– groups is signaled by a signal corresponding to a chemical shift of 22.01 ppm. The magnitude of these signals, other than for pure PET, which are multiples of signals from PET, indicate that this is not an admixture but rather a defined copolymer.

This information regarding the chemical structure of the PET used in the production of the preform indicates that the values known from the literature for pure PET of amorphous phase density $\rho_{a}^{u}=1.335\;g/{\mathrm{cm}}^{3}$ [6] and crystalline phase density $\rho_{c}=1.455\;g/{\mathrm{cm}}^{3}$ [6] should be considered only as a starting point for further considerations regarding the calculation of the relaxation of the amorphous phase.

## 3. Determining the Sample Size

Before statistical analysis of the influence of the blow mold temperature (cold mold or hot mold) and hot filling methods (Series “A”, no annealing; Series “B”, free annealing; and Series “C”, bath annealing), the minimum sample size for not exceeding the acceptable maximum error was specified, but only for the hot mold SBM process. Due to the smaller number of process parameters and the easier to stabilize SBM technological process with a cold mold, the number of samples for the cold mold was taken as the same amount as for the hot mold. The results determining the sample size for the hot mold SBM process are summarized in in Tables 1–4.

### 3.1. Tests Determining the Sample Size for Measuring the Thickness and Dimensions of Blow Kinetics Marks for One Bottle, n₁ and n₂, Respectively

The method of statistical analysis of the original dataset for three randomly selected bottles from a population of 30 bottles of Series “A” for the hot mold is described in the first part of the paper [5]. Table A1 presents the results of the thickness sample size for each measuring point and the dimensions of each blow kinetics mark of each bottle in the measurement series. The estimated standard deviation in the population for one bottle ($\sigma_{p}$) was defined as the maximum standard deviation for three randomly selected bottles from the population of 30, where n₁ is the sample thickness measurement size for each measuring point and n₂ is the sample size for measuring the dimensions of each blow kinetics mark.

### 3.2. Tests Determining the Sample Size for Thickness Measurements and Dimensions of Blow Kinetics Marks for the Entire Measurement Series “A” (Number of Bottles in the Series), n₃ and n₄, Respectively

A method of conducting statistical analysis of the original dataset for 30 bottles manufactured in Series “A” of hot molding is described in the first part of the paper [5]. Table A2 presents the results of the sample size for the measurement series (number of bottles in the measurement series) for measuring the thickness of each measurement point (n₃) and the dimensions of each blow kinetics mark (n₄).

### 3.3. Tests Determining the Sample Size for Pressure Resistance and the Weight of the Bottle Filled with Water for the Entire Measurement Series “A”, “B”, and “C” (Number of Bottles in the Series), n₁₁ and n₁₂, Respectively

A method of conducting statistical analysis of the original dataset for the 30 bottles manufactured in Series “A”, “B”, and “C” of hot molding is described in the first part of the paper [5]. Table A3 presents the results of the sample size for the measuring Series “A”, “B”, and “C” (number of bottles in the measuring series) for measuring the burst pressure of the bottle (n₁₁) and the weight of the bottle filled with water (n₁₂).

### 3.4. Tests Determining the Sample Size for Measurements of the Degree of Crystallinity by the DSC Method and the Density of the Material in a Gradient Column Method for the Entire Measurement Series “A”, “B”, and “C”

A method of conducting statistical analysis of the original dataset for the 30 bottles manufactured in Series “A”, “B”, and “C” of hot molding is described in the first part of the paper [5]. Table A4 presents the results of the sample size for the measuring Series “A”, “B”, and “C” (number of bottles in the measuring series) for measuring the degree of crystallinity by the DSC (differential scanning calorimetry) method and the density of the material in a gradient column.

Table A4 shows that the number of bottles required to not exceed the maximum acceptable error is too large to carry out a comparative analysis of the effect of the blow mold temperature and hot filling method on the measurement of the material density by the gradient column and the degree of crystallinity by DSC. In this case, the sample size was determined by the minimum sample size for which it is possible to carry out statistical analysis, and it was assumed that, from the population of 15 bottles produced for each Series “A”, “B”, and “C” for cold and hot blow molds, three bottles would be drawn at random, on which degree of crystallinity and density tests would be carried out with a single measurement repetition. Therefore, only the change in the microstructure of the bottle relative to the preform will be analyzed. In addition, it should be emphasized that, in this case, the analysis of the degree of crystallinity, density, and orientation of the amorphous phase will constitute only qualitative research.

### 3.5. Sample Size for the Entire Research (Number of Bottles for All Series) of Thickness Profile, Kinetics of Blowing, Pressure Resistance, and Thermal Stability

Analyzing the number of bottles required in each Series “A”, “B”, and “C” for hot molding in order not to exceed the acceptable error, it follows from the results in Table A2, Table A3 and Table A4 that the maximum number of bottles for the series is n_max_ = 15. Therefore, for each measuring Series “A”, “B”, and “C” for both the hot and cold molds, 15 bottles for each series were accepted. Then, from this population of 15 bottles produced for the relevant study, the adopted number of bottles specified in Table A2 and Table A3 was drawn. The above results relate to the determination of the sample size for each Series “A”, “B”, and “C” of the hot mold. For Series “A”, “B”, and “C” of the cold mold, the same sample sizes for the relevant tests were adopted, due to the authors’ experience that it is more difficult to stabilize the production process of hot fill bottles (for a hot mold) than cold fill ones (for a cold mold).

For a hot mold from a population of 30 bottles previously made, a specific number of bottles were drawn using a random number generator, thus, from the population of 30 in each Series “A”, “B”, and “C”, 15 bottles were drawn. From this population of hot fill bottles and from the population of cold fill bottles using the same generator, bottles were drawn for each of the feature measurements (specified in Table A2 and Table A3).

### 3.6. Investigation into the Stabilization of the SBM Process with a Cold Mold and with a Hot Mold

To determine the stabilization of the SBM process with hot and cold molds, a test was done for the homogeneity of variance and a test for the significance of differences between the mean of measurements for each bottle and the mean value for the whole Series “A” for the thickness measurements and dimensions of the blow kinetics determination of each bottle Series “A” separately. The homogeneity of variance was determined by the Levene test, while the significance of differences between the average for each bottle and the average for all bottles (average for the whole Series “A”) was determined by the most conservative post-hoc Scheffé test. It was assumed that, if, for 90% of bottles, there are no significant differences between the measurements of each bottle and the average value for the whole series, then the difference between the average for the bottles in the whole series is insignificant. The method of testing is summarized in the first part of the paper [5], according to the designation: (*) data used to determine the process stability in terms of the thickness profile; and (**) data used to determine the process stability in terms of the blow kinetics.

It should be emphasized that the variance homogeneity test only makes it possible to determine whether, at the time of measurement, the measurement result (dependent variable) was also influenced by another uncontrolled factor than the independent variable. If the homogeneity of variance occurs for the tested measuring groups, it means that all uncontrolled factors randomly influenced the dependent variable, whereas, if the homogeneity of variance is not maintained, it means that there was some uncontrolled factor that affected the dependent variable in a non-random way. Since the uniformity of variance may or may not be maintained, and the differences between means may or may not be significant, there are four possible combinations of the results of these two tests. The interpretation of the test results for stabilizing the SBM process is summarized in Table 1.

Table A5 summarizes the results of the Levene and post-hoc Scheffé tests for measurement of the thickness at the measurement points (with a repeat of three and five times the measurement according to Table A1) and for measurement of the dimensions needed to calculate the blow kinetics coefficients at the measurement points (with a repeat of five times the measurement according to Table A1) for 30 bottles produced in the SBM process with the hot mold and for 15 bottles produced in the SBM process with the cold mold. Table A5 shows that the SBM process due to the blow kinetics and the thickness profile can be considered stabilized (the exception is the measurement of the blow kinetics dimensions p2-II and f-IV for the cold blow mold). Moreover, only for measuring the dimensions p3-III and f-II for a cold mold can the assumption be made that it is possible to assume the homogeneity of variance between bottles in the measurement series. It follows that the process had an uncontrolled factor affecting the measured thickness profile and blow kinetics dimensions, but it did not affect the stability of the SBM process. The means of measurements for each bottle statistically significantly different from the mean value for the whole Series “A” were removed from the dataset (the numbers of these bottles are given in brackets).

## 4. Statistical Analysis and Interpretation of Measurement Results

Table 2 shows the plan for one one-factor bivalent (1x2, “number of factors”x“number of levels of factor”) experiment for the SBM process, where the first value of the factor was the cold mold temperature and the second value of the factor was the hot mold temperature for Series “A” of the hot filling method. Studies were done on the thickness profile and blow kinetics for this plan.

Table 3 shows the plans for six one-factor bivalent (1x2) experiments for the SBM process with the hot fill process, where the first value of the factor was the preform feature and the second value of the factor was the bottle feature produced in the SBM process with a combination of two mold temperatures (cold or hot) and three hot filling methods (“A”, “B”, or “C”). Studies on the degree of crystallinity, density, and relaxation of the amorphous phase were performed for each of the six plans.

Table 4 shows the plans for nine one-factor bivalent (1x2) experiments for the SBM process for the hot fill process. Studies on the pressure resistance and thermal stability were performed. In addition, three two-factor bivalent plans were adopted, in which a linear model with two main effects and one two-factor interaction effect was implemented separately. The plans of three two-factor bivalent (2x2) experiments for the mold temperature and three combinations of hot filling, i.e., without hot filling (“A”) and free annealing (“B”), without hot filling (“A”) and bath annealing (“C”), and with free annealing (“B”) and bath annealing (“C”), are presented in Table 5. For each plan, bottle pressure resistance tests were carried out and for one, additional thermal stability tests. 

For every plan, the power of ANOVA statistical test was calculated.

### 4.1. DOE Analysis of the Impact of the SBM Process (Blow Mold Temperature) on the Change of Thickness Profile and Kinetics of Bottle Blowing—NoE: 1

**Figure 2:**
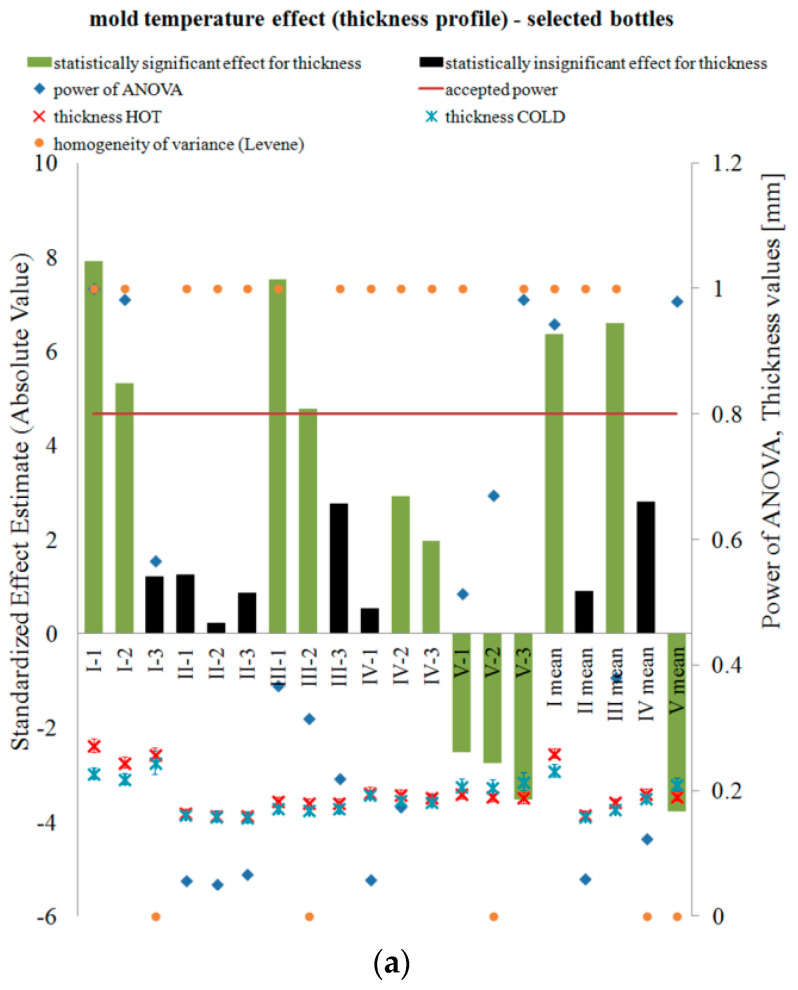

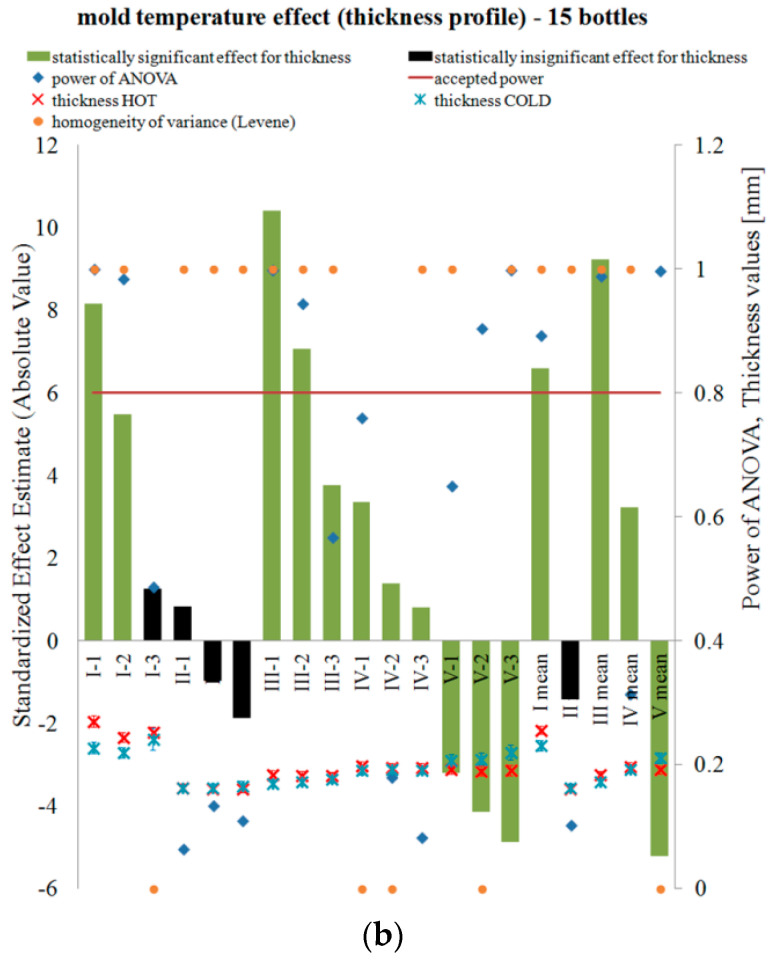
The results of testing the impact of blow mold temperature in a single-factor plan on the bottle thickness profile, along with the results of the analysis of the power of the tests carried out, Levene test of homogeneity of variance results (0, no homogeneity; 1, homogeneity occurs), as well as the average thickness and measurement uncertainty: (**a**) for selected bottles in accordance with Table A2; and (**b**) for all 15 bottles in the Series “A”. Effects that do not meet the adopted assumption with a 5% probability of making a first type error are marked in black.

**Figure 3:**
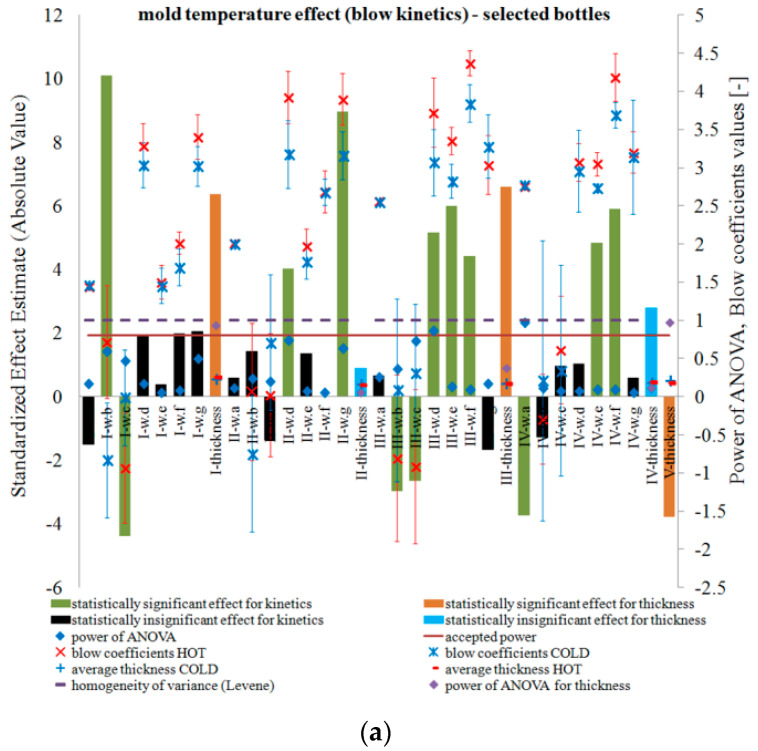

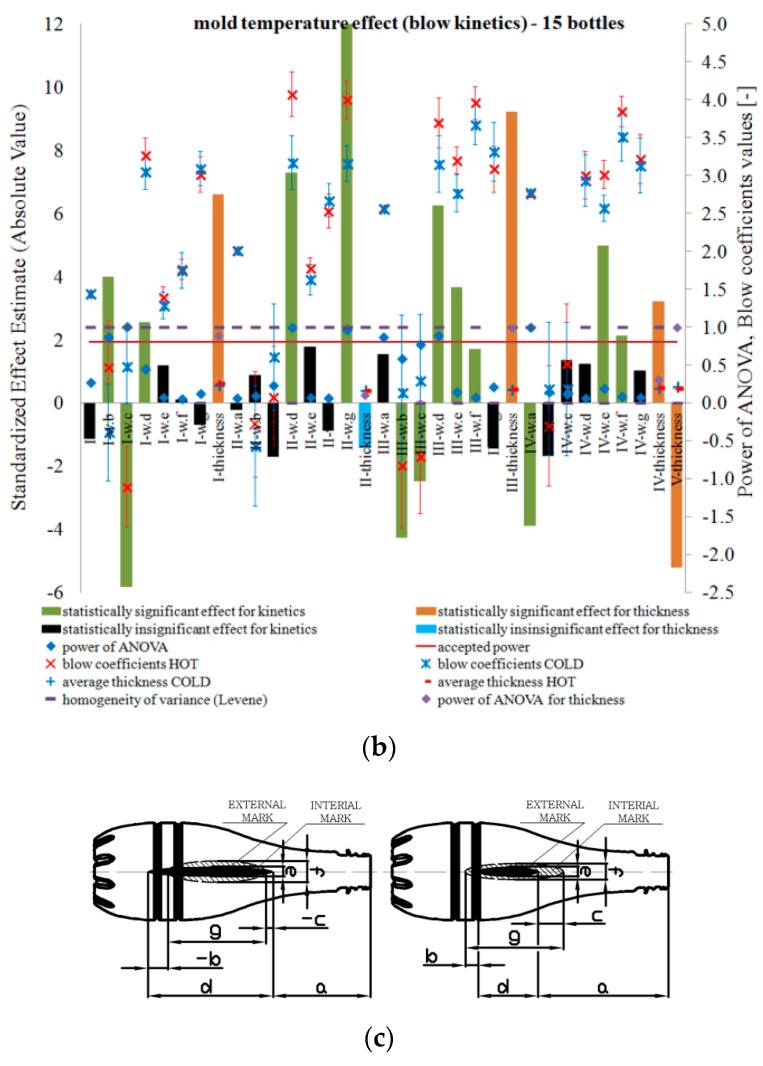
The results of testing the impact of the blow mold temperature in a single-factor plan on the blow kinetics coefficients, along with the results of the power analysis results of the tests carried out, Levene test of homogeneity of variance results, (0, no homogeneity; 1, homogeneity occurs), as well as the value of the average coefficients and measurement uncertainty: (**a**) for selected bottles in accordance with Table A2; and (**b**) for all 15 bottles in the Series “A”. Effects that do not meet the adopted assumption with a 5% probability of making a first type error are marked in black and blue; (**c**) illustration of the location of dimensions “a, b, c, d, e, f, g” of markers on the bottle needed to calculate the corresponding coefficients of blow kinetics “w.a., w.b, w.c, w.d, w.e, w.f, w.g” (as it is described in first part of the paper [5]).

**Figure 4:**
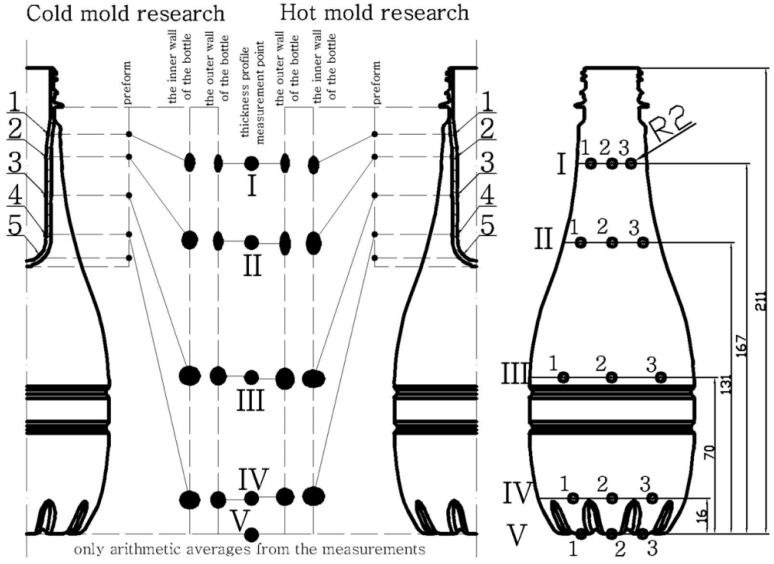
Graphic display of the displacement and deformation of the blow kinetics marks which are measured by the blow kinetics coefficients (Figure 3) together with the explanation of determining the location of the thickness measurement points. Only arithmetic averages from the measurements were taken into account, without taking into account the measurement spread.

Figure 2 and Figure 3 show the results of testing the impact of the blow mold temperature in a one-way plan, respectively, on the thickness and coefficients of blow kinetics for selected bottles in accordance with Table A2 (a) and all 15 bottles in Series “A” (b). The figures also include the results of the analysis of the power of the analyses carried out, the Levene test homogeneity of variance results, and the mean values of the analyzed quantities along with the measurement uncertainty calculated in accordance with the description in the corresponding first part of the paper [5]. When determining the blow kinetics coefficients, it was noticed that the V mark made on the preform did not change its dimensions or position on the bottle, which was caused by the cooling of the preform gate area with a stretching rod. The remaining markers shifted and deformed, which is graphically shown in Figure 4.

Figure 2 shows that, regardless of the temperature of the blow mold, the greatest thickness is obtained around the shoulder part of the bottle, and the smallest in the vicinity of the bottle label. The thickness of the bottle near the bottom is greater than for the label area but only slightly, compared to the difference between the thickness around the label and around the shoulder. Most likely this has to do with the shape of the bottle being analyzed and the narrow shape of the arms. 

In addition, Figure 2 shows that the higher is the temperature of the blow mold, the greater is the thickness of the shoulder area, label, and top of the bottom, while it decreases for the bottom of the base. This can be explained by the fact that, in a cold blow mold, the material of a blown bottle is cooled by the air between the surface of the mold and the surface of the blow bottle, while, in a hot blow mold, the material of a blown bottle is heated up even more strongly by the air (enclosed between the surface of the blow mold and the surface of the blown bottle), as a result of which, in the initial stage of blowing, even during the movement of the stretching rod, the material is blown more quickly (in relation to the position of the stretching rod) for a hot mold than for a cold mold and more material remains around the shoulder and label. This is indirectly demonstrated by the influence of the blow mold temperature on the change of the coefficient $∆w_{a}$ for the IV marking, where the marking has moved towards the neck for the hot blow mold, which could have been caused by faster blowing of the bottle around the shoulders and label in the case of the hot blow mold. For Determinations I–III, there is no statistical effect of the blow mold temperature on the change of the coefficients $∆w_{a}$.

It should be emphasized that the results of the analysis of variance homogeneity for the thickness and blow kinetics coefficients are practically satisfied for all tests. This means that the measurement result was not affected by any uncontrolled factor and at the statistical level it can be clearly determined what was measured. 

Figure 3 and Figure 4 show that, irrespective of the mold temperature, the smallest circumferential enlargement of the markers during blowing occurs at Point I and increases rapidly towards the bottom. However, for Point III, the peripheral magnification of the markers is greater than for the Point IV markers. This is due to the “pear” shape of the bottle. In addition, Figure 3 shows that the circumferential deformation of the inner markers is greater than the circumferential deformation of the outer markers. This is due to the fact that, during blowing the thickness decreases, thus the relative circumferential displacement of the inner wall is greater than the outer wall of the bottle. It also follows that, in the movements of the material in the bottle wall during blowing, the material close to the inner wall has a greater share than the material close to the outer wall before the preform material reaches the mold. The analysis of the influence of the mold temperature on the change of the axial and circumferential dimensions of the external and internal markings is strongly distorted by the strongly changing shape of the blow mold depending on the axial position of the analyzed mark. Therefore, this analysis is not carried out. In other words, the coefficients $∆w_{d}$, $∆w_{e}$, $∆w_{f}$, and $∆w_{g}$ are not independent of each other for different mold temperatures because they strongly depend on the factor $∆w_{a}$.

However, it is possible to perform an analysis of the relationship between the shape of the external markings and internal markings, as well as the effect of the blow mold temperature on changing this relationship. For the hot mold, the analysis of the location of the external markings relative to the position of the internal markings shows that, for Point I, the internal markings move towards the bottom relative to the position of the external mark—as around Point I the inner wall of the bottle moves relative to the outer wall of the bottle towards the bottom. This is due to the fact that the outer wall encounters resistance (friction) when it comes into contact with the wall of the blow mold. For Point II, the internal marking does not move relative to the external marking (the internal marking is slightly moved towards the bottom relative to the external marking). For Point III, the outer marking is shifted towards both the bottom and the thread relative to the location of the inner marking, although more towards the bottom than towards the thread. However, for Point IV markings, the outer mark moves towards the bottom relative to the position of the inner mark. To sum up, it can be stated that, in the case of a hot blow mold, for the shoulder of the bottle, the inner wall slips against the outer wall towards the bottom, while for the bottom of the bottle the opposite is true, and the inner wall slips against the outer wall towards the bottle thread. The trend changes around Point III. This is understandable behavior of the bottle wall during blowing. From this gradient of axial displacement in the thickness direction, it follows that, for the shoulder area, the outer wall of the bottle “meets” the blow mold wall in the direction “from the thread to the bottom”, while, for the bottom, the outer wall of the bottle “meets” the blow mold wall in the direction “from the bottom to the thread”.

The behavior of the internal markings relative to the external form is different for a cold blow mold. Thus, for determinations I–IV, the internal designation is shifted towards the thread relative to the external designation and much more strongly for Designation II than Designation I, while, for Designations III and IV, practically no shift occurs (it is not statistically significant). This behavior of the material indicates a rather surprising movement of the material in the wall of the blown bottle. Regardless of the temperature of the blow mold, the inner wall of the bottle was more circumferentially deformed than the outer wall of the blown bottle. Thus, as a result of orienting the macromolecules, it strengthened more strongly as a result of blowing than the outer wall. Thus, during the blow, it is “easier” to move from the outside of the bottle’s shoulder towards the label (and from around the label towards the bottom). However, the difference between a cold and hot mold occurs after the wall of the blown bottle comes into contact with the wall of the blow mold. For a cold blow mold, the bottle material is immediately cooled to below the softening point, preventing further movement of the inner wall relative to the outer. On the other hand, for a hot blow mold, after the outer wall of the bottle comes into contact with the wall of the blow mold, the material of the bottle is not cooled, but the outer wall will encounter resistance (by friction) without moving further, while the material from the inner wall of the bottle is constantly moving as a result of being “pulled” by the material being blown around the label and bottom.

The movement of the internal markings relative to the external ones for Points I–III depending on the temperature of the mold is statistically significantly different. However, the effect of the blow mold temperature on the displacement of the internal markings relative to the external ones for Point IV is statistically insignificant.

However, the power of ANOVA testing is very low for the thickness measurement and blow kinetics. Only for the analysis of the impact of the blow mold temperature on the thickness around the shoulder (Point I) and base (Point V), the test power is acceptable on the basis of the number of bottles specified in Table A2, while for the part of the label and blow kinetics coefficients the number in the series of bottles specified in Table A2 is insufficient in terms of statistical test power. Increasing the number of bottles to 15 for all tests only slightly increased the power of the statistical tests for analyzing the effect of the blow mold temperature on the thickness profile and blow kinetics. It follows that, in terms of the test power and thus the likelihood of failure of a second type of error, more bottles are needed in the test series than 15, especially for the analysis of the blow kinetics. It follows also that any analysis that did not show the effect of the blow mold temperature on a dependent variable is most likely erroneous; this effect does occur, but it is not known what effect it is.

### 4.2. DOE Analysis of the Impact of the SBM Process and Hot Fill Process on the Change of Density, Degree of Crystallinity and Orientation of the Amorphous Phase of the Bottle Material Relative to the Preform Material—NoE: 2–7

**Figure 5:**
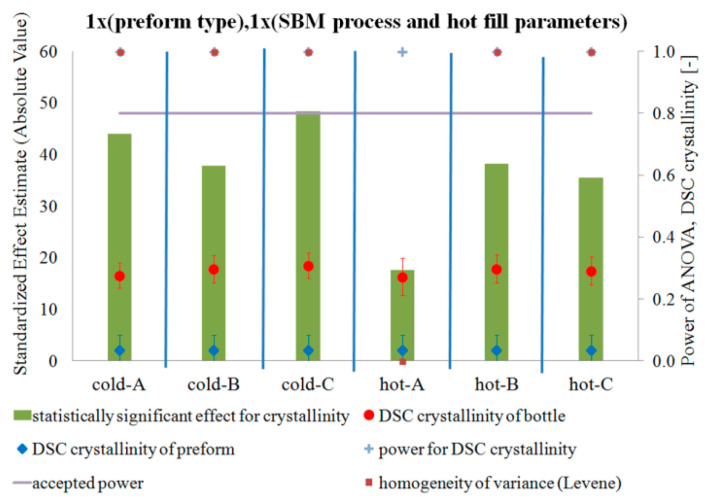
The results of testing the impact of the blow mold temperature and hot filling method in a one-way test plan on the degree of crystallinity of the bottle material relative to the preform material, together with the results of the analysis of the power of the tests carried out, the results of the homogeneity of variance (Levene test), and the value of the average degree of crystallinity and measurement uncertainty.

**Figure 6:**
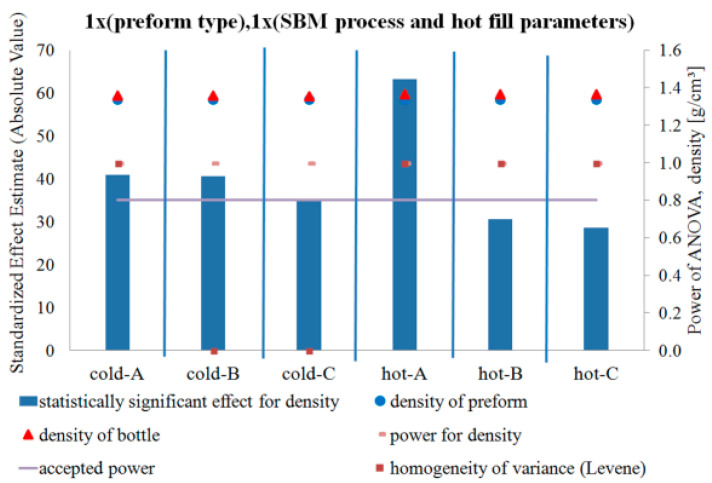
The results of testing the impact of the blow mold temperature and hot filling method in a one-way test plan on the density of the bottle material relative to the preform material, together with the results of the analysis of the power of the tests carried out, the results of the homogeneity of variance (Levene test), and the value of the average density and measurement uncertainty.

**Figure 7:**
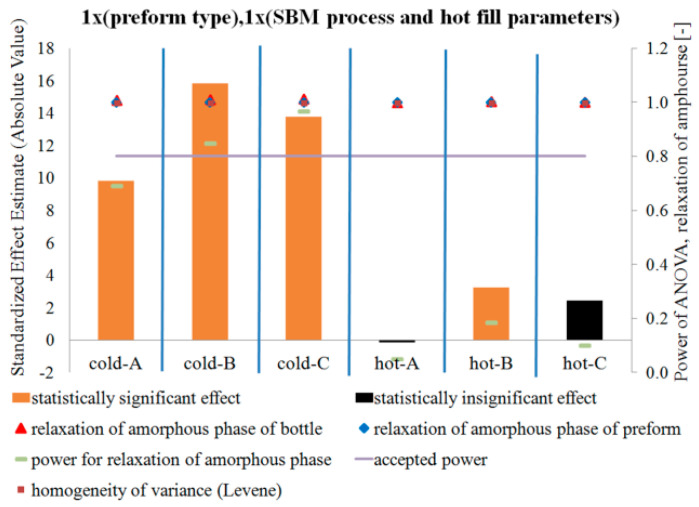
The results of testing the impact of the blow mold temperature and hot filling method in a one-way test plan on the relaxation of the amorphous phase of the bottle material relative to the preform material, together with the results of the analysis of the power of the tests carried out, the results of the homogeneity of variance (Levene test), and the value of the average relaxation of the amorphous phase and measurement uncertainty.

**Figure 8:**
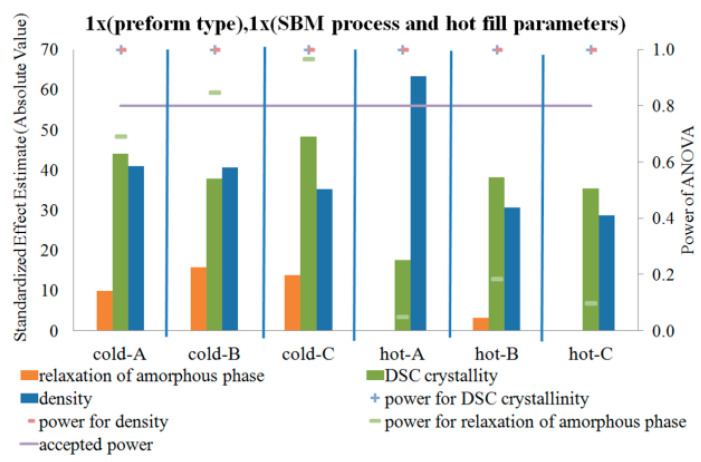
List of statistical analysis results including the power of tests shown in Figure 5, Figure 6 and Figure 7.

Figure 5, Figure 6 and Figure 7 show the influence of the blow mold temperature and hot filling method in a one-way test plan on the degree of crystallinity, density, and relaxation of the amorphous phase of the bottle material relative to the preform material, respectively. The figures (and all the figures described below in the paper) also include the results of the analysis of the power of the analyses carried out, the results of the homogeneity of variance (Levene test), and the mean values of the analyzed quantities along with the measurement uncertainty (calculated in accordance with the description in the corresponding first part of the paper [5]). Figure 8 summarizes the results shown in Figure 5, Figure 6 and Figure 7.

Figure 5 and Figure 6 show that the SBM process and the filling process significantly affected both the degree of crystallinity and the density of the bottle’s material relative to the preform material—both values increased. However, Figure 7 shows that the SBM process and the hot filling process did not always change the relaxation of the amorphous phase of the bottle material relative to the preform material. For the SBM process with the hot mold and without the hot filling process (Hot-A) and the SBM process with the hot mold and annealing in the bath after hot filling (Hot-C), there was no statistically significant change in the degree of relaxation of the amorphous phase, and even, for the SBM process with the hot mold and free annealing after a hot filling (Hot-B), the change in the degree of relaxation of the amorphous phase of the bottle material relative to the preform material is significantly less than for the Cold-mold SBM process. However, it should be very strongly noted that the test power for the Hot-A, Hot-B, and Hot-C cases is close to zero, thus we make a practically certain Type II error in assuming the null hypothesis is true when it is incorrect (in other words, the conclusion that for the Hot-A and Hot-C series the SBM process had no effect on the degree of relaxation of the amorphous phase of the bottle’s material relative to the preform material is with more than 90% probability incorrect). It is also worth noting that the homogeneity of variance between the measurements of the degree of crystallinity of the preform material and the bottle material for Hot-A is not maintained, while it is already preserved for all the other series. Perhaps for the Hot-A series there was some uncontrolled factor when testing the degree of crystallinity of the bottle (Figure 7 shows that only for this series the amorphous phase relaxation was lower for the hot mold than for the cold one, but this effect was not statistically significant with a 5% assumption, making an error of the first type). In addition, the lack of homogeneity of variance occurs when testing the density for the Cold-B and Cold-C series. However, it is not known what could be the reason for this, although, due to many factors affecting the properties of the bottle, the bottle is characterized by very heterogeneous microstructure properties, even in the area of the cut sample with dimensions 1 cm × 1 cm. Due to the very strong heterogeneity, the number of repetitions of the measurement of the bottle’s microstructure characteristics should be increased.

The increase in the degree of crystallinity and density of the bottle material relative to the preform material after the SBM process is understandable and obvious (as a result of heating the preforms in the heating oven and subsequent rapid deformation, the material crystallized and thus increased in density). It is interesting, however, that, in every case of a statistically significant change in the degree of relaxation of the amorphous phase, the degree of relaxation of the amorphous phase also increased after the SBM process and hot filling—i.e., the density of the amorphous phase of the bottle’s material is less than the density of the amorphous phase of the preform.

The above phenomenon is very puzzling, because it would suggest a decrease in the nematic and smectic mesophases of the amorphous phase in the bottle material relative to the preform material, which is not confirmed anywhere in the literature. However, another phenomenon can be found in the literature that may explain this specific behavior of PET during the SBM process. This is the phenomenon of microcavitation [7] at the border of the crystalline phase and the amorphous phase during rapid deformation of this material—increasing the free volume of the material in the area of the amorphous phase at the border with the crystalline phase, as a result of which the density of the non-crystalline phase in the volume of the sample decreases, which is ideally shown in Figure 10. Microcavitation processes occur, but the PET chains do not crack (the breaking strength of covalent bonds in a carbon-based chain is 60–100 GPa, which is a gigantic value compared to the strength limit of ordinary steel, which is 0.3–0.6 GPa). It must be strongly emphasized that, if the free volume statistically significantly changes during the SBM process, correlating the density of the non-crystalline phase with the degree of relaxation of the amorphous phase loses any sense. The free volume of the preform and bottle should be measured, e.g., by positron annihilation testing.

However, in the literature it was found that a decrease in the density of PET material relative to the density of the amorphous phase may occur, not as a result of stretching the material, but as a result of immersion of the unstretched PET for 30 min in acetaldehyde. The authors of [8] showed that the immersion of unstretched PET for 30 min in acetaldehyde causes:

- a reduction of the amount of trans conformation of two carbonyl groups adjacent to the benzene ring;
- an increase of the amount of ordered lamellar crystals (increasing the trans conformation of the glycol group);
- an increase of the amount of perpendicular arrangement of the carbonyl group and benzene ring relative to each other; and
- a decrease in density perpendicular to the plane (100).

The above statements are probably connected with a reduction in the structural packing of PET chains. A similar phenomenon may occur in the samples tested because it is not pure PET. The compound, which is additionally found in the samples, can cause similar changes in conformation and thinning of the system, and thus a decrease in the density of the material.

**Figure 9:**
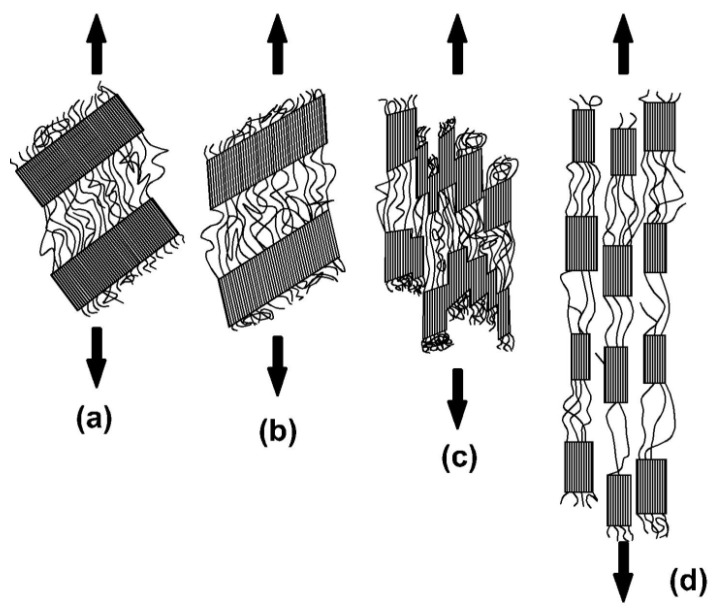
Stages occurring during deformation of a semi-crystalline linear material with fibrillar crystal structures [9]: (**a**) stretching of inter-lamellar amorphous areas; (**b**) deformation of lamellas as a result of stretching, which results in recrystallization of crystalline parts of fibrils; (**c**) disintegration of lamellas as a result of high tangential forces from related macromolecules; and (**d**) formation of microscopic crystal structures in the form of fibers.

The reduction in the density of the material relative to the density of the non-oriented amorphous phase can also be explained in another way. During stretching of the initially crystalline material, the crystallinity of the material may decrease in some areas due to the disturbed structure while in other places the crystallinity may increase. In addition, the crystal structures during the deformation of the sample can rotate and tear, as illustrated in Figure 9 [9]. Individual phases of disruption of fibrillar structures shown in Figure 9 according to the authors of [9] are as follows: (a) stretching of inter-lamellar amorphous areas; (b) deformation of lamellas as a result of stretching, which results in recrystallization of crystalline parts of fibrils; (c) disintegration of lamellas as a result of high tangential forces from related macromolecules (covalent forces in the main chain are stronger than van der Waals forces between macromolecules in lamellar structures, which is why crystal structures are broken, but not the chains in the amorphous region); and (d) formation of microscopic crystal structures in the form of fibers. What is more, when the local oriented amorphous area crystallizes (increases the order), the number of chains decreases in the surrounding area and thus the density of the surroundings (because some of the chains from this area have been introduced into the crystallizing area), which increases the disorder of the chains around the crystalline area (colloquially speaking, disorder decreases, i.e., the entropy of the environment increases). This phenomenon has been observed by several researchers (e.g., [10]). The authors of [11] claimed that, in the case of strain-induced crystallization caused by deformation of PET material, the crystallization nuclei with a triclinic PET structure strongly interact with the surrounding deformed chain network, which means that the relaxation processes of these chains also affect the orientation of the crystallization nuclei (and the further orientation of the crystalline phase).

**Figure 10:**
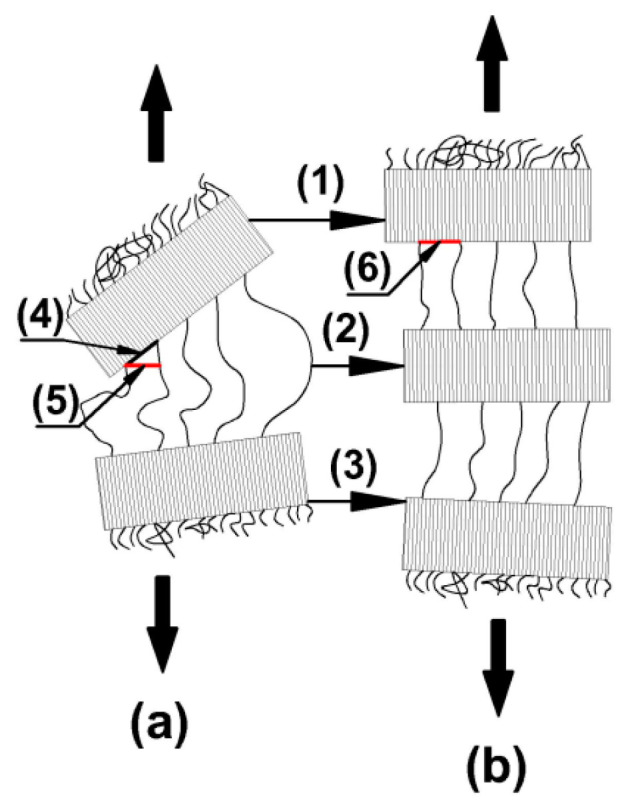
Hypothetical considerations of the influence of crystallite turnover on the behavior of the free volume on the border of crystallites with the amorphous phase, as a result of which the density of the oriented amorphous phase may decrease relative to the non-oriented amorphous phase of the semi-crystalline polymer (detailed description in the text), (**a**) stretching of inter-lamellar amorphous regions; (**b**) crystallization of the oriented amorphous phase to the form of crystallite and an increase in the free volume in the area of the boundary of the crystalline phase with the amorphous on the rotation of crystallites as a result of deformation (cavitations in the volume of amorphous phase of the material).

Figure 10 shows hypothetical considerations of the effect of crystallite rotation on the behavior of the free volume at the boundary of crystallites with the amorphous phase. In Figure 10, the individual markings mean: (a) stretching of inter-lamellar amorphous regions; (b) crystallization of the oriented amorphous phase to the form of crystallite and an increase in the free volume in the area of the boundary of the crystalline phase with the amorphous on the rotation of crystallites as a result of deformation (cavitations in the volume of amorphous phase of the material); (1 and 3) rotation of crystallites; (2) crystallization of the oriented amorphous phase as a result of deformation (strain- induced crystallization); (4) distance between adjacent chains coming out of the crystallite on the border of the crystalline phase with the amorphous phase before the stretching process; (5) distance between adjacent crystallites in a direction to the final direction of the boundary of the crystalline phase with the amorphous phase (i.e., in the direction of the boundary of the crystalline phase with the amorphous phase after the rotation of the crystallite as a result of stretching); and (6)distance between adjacent chains going out of crystallite on the border of the crystalline phase with the amorphous phase after the stretching process. As the hypothetical considerations show, as a result of the crystallite rotation in the amorphous phase, the distance increases between adjacent chains coming out of the crystallite at the border of the crystalline phase with the amorphous, and thus the free volume increases, which results in a decrease in the density of the oriented amorphous phase relative to the non-oriented.

What is more, it is worth emphasizing that numerical simulations of rubber state models with nematic microstructure show that, during uniaxial deformation of such material, there is a simultaneous transverse contraction of the sample that is smaller than the contraction of the microstructure induced by the orientation of the particles in the nematic order [12]. This may be due to the fact that microcavity effects occur during the deformation of the material and that these are associated with the orientation of the microstructure. The intensification of the cavitation process in the purified polypropylene samples can be explained by changes in the amorphous phase, i.e., the changes in free volume arising from the elimination of low fractions and soluble additives. An increase in free volume was probed with positron annihilation lifetime spectroscopy. The intense formation of cavitation pores in purified polypropylene proves that the initiation of cavitation in polypropylene has a homogeneous nature (the homogeneous nucleation of cavitation), as shown by [7]. These authors predict similar behavior in other crystalline cavitating polymers and associate this with the extraction of additives and the low molecular weight fraction.

### 4.3. DOE Analysis of the Influence of Blow Mold Temperature and Hot Fill Method on Pressure Resistance and Thermal Stability of the Bottle in a Single-Factor Plan—NoE: 8–16

**Figure 11:**
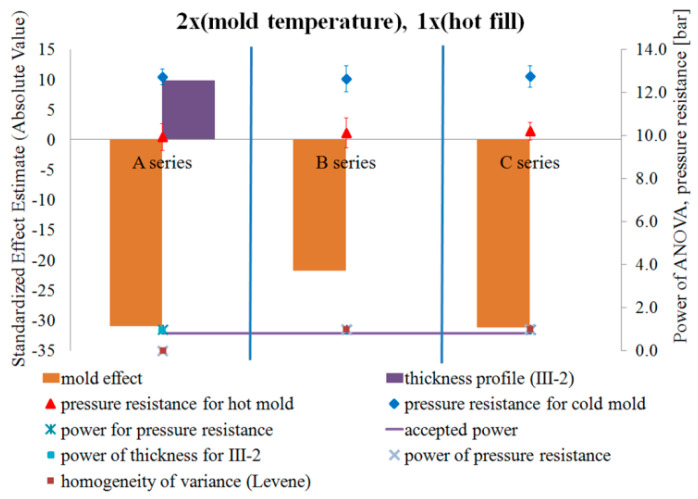
The results of testing the impact of the blow mold temperature in a single-factor test plan on the pressure resistance of the bottle material, together with the results of the analysis of the power of the tests carried out, the results of homogeneity of variance (Levene test), and the value of the average pressure resistance and measurement uncertainty (NoE: 8–10).

**Figure 12:**
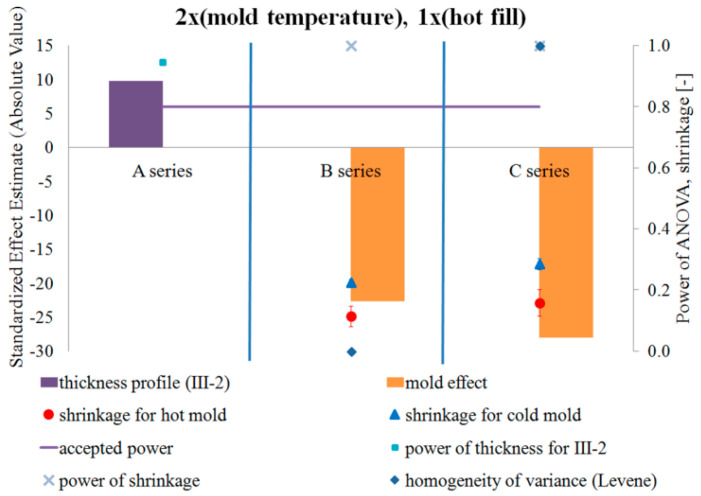
The results of testing the impact of the blow mold temperature in a single-factor test plan on the thermal shrinkage of the bottle material, together with the results of the power analysis of the tests carried out, the results of the homogeneity of variance (Levene test), and the average thermal shrinkage and measurement uncertainty (NoE: 8–10).

Figure 11 and Figure 12 show the results of testing the impact of the blow mold temperature in a single-factor plan on the pressure resistance and thermal shrinkage, respectively. Figure 11 and Figure 12 also show the results of the blow mold effect on thickness at Point III, i.e., where the cracking began during the pressure resistance tests.

Comparing the pressure resistance and thermal stability (thermal shrinkage) of a bottle made in a cold and hot blow mold for the case before the hot fill process (just after production) and after the hot fill process (after the free annealing or bath annealing), it follows that:

- Both without a hot filling and after a hot filling, the pressure resistance always decreases for a hot blow mold compared to a cold blow mold.
- A hot blow mold strongly reduces bottle shrinkage (increased thermal stability) relative to a cold blow mold.

**Figure 13:**
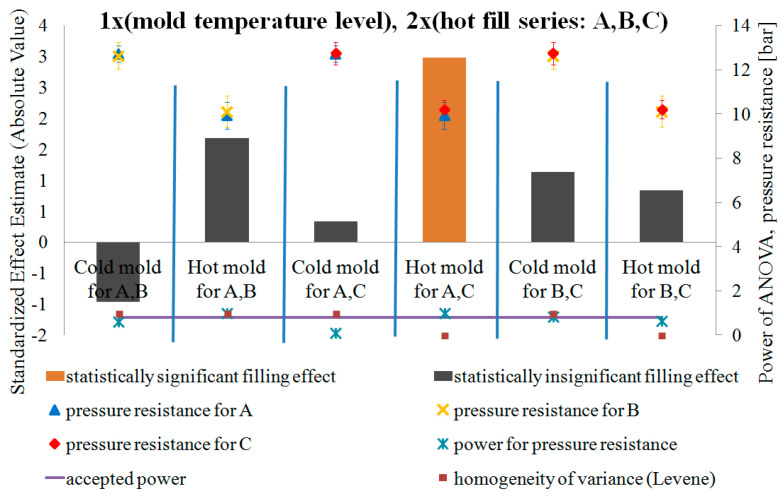
The results of the research on the impact of the hot filling method in a single-factor test plan on the pressure resistance of the bottle material, together with the results of the analysis of the power of the tests carried out, the results of the homogeneity of variance (Levene test), and the value of the average pressure strength and measurement uncertainty—black indicates the effects that do not meet the adopted assumption of 5% probability of committing the first type of error (NoE: 11–16).

**Figure 14:**
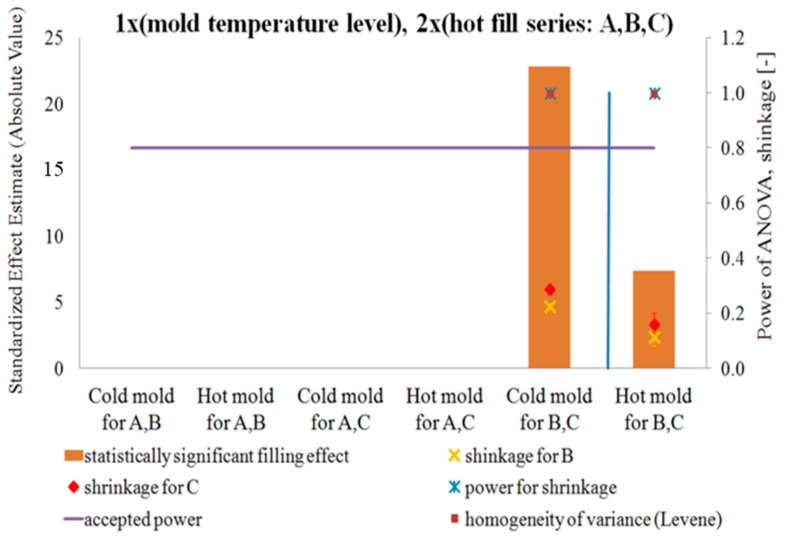
The results of the research on the impact of the hot filling method in a single-factor test plan on the thermal shrinkage of the bottle material, together with the results of the analysis of the power of the tests carried out, the results of the homogeneity of variance (Levene test), and the average value of the thermal shrinkage and measurement uncertainty (NoE: 15 and 16).

Figure 13 and Figure 14 show the results of the study on the impact of the hot filling method in a single-factor plan on the pressure resistance and thermal shrinkage of the bottle material, respectively.

Comparing the pressure resistance and thermal stability (thermal shrinkage) of the bottle material before the hot fill process (just after production) and after the hot fill process (after the free annealing and bath annealing process) separately for cold and hot blow molds, it results that:

- The method of annealing after hot filling did not have a statistically significant effect on the change in pressure resistance, regardless of the temperature of the blow mold.
- The method of annealing after a hot filling (free or in a bath) has a statistically significant effect on the thermal shrinkage, regardless of the blow mold (in both cases, the shrinkage after the bath annealing is much greater than the shrinkage after free annealing). The thermal shrinkage of the bottle annealed in the bath is greater than the thermal shrinkage of the free-annealed bottle, and the thermal shrinkage also increases more rapidly for a cold blow mold than for a hot blow mold. In addition, the power of the tests is very large.
- Surprising results were obtained for pressure resistance. For the cold blow mold, the hot fill process and annealing process (free or in bath) during the hot fill process do not have a statistically significant effect on the pressure resistance. Only in the case of a hot blow mold, bath annealing of the bottle in a hot fill process (Series “C”) increases the pressure resistance compared to a bottle without the hot fill (Series “A”). However, it should be emphasized that the ANOVA analysis result when comparing Series “A” and “C” for a cold blow mold has a practically 100% probability of making a Type II error. Thus, most likely, also for a cold blow mold, bath annealing of the bottle increases the pressure resistance in a statistically significant way compared to a bottle without a hot fill (which results from the analysis of the arithmetic mean of the measurements). It follows that the pressure resistance increases after bath annealing in a hot fill process (compared to Series “A”) more strongly for a hot blow mold than for a cold blow mold, which is inversely correlated with the increase in thermal shrinkage. Thus, the change in pressure resistance can also be influenced by the change of size of the bottle (the greater is the shrinkage of the bottle and thus the smaller is the diameter of the bottle, the greater is the pressure resistance of the bottle).

It should be very strongly noted that, after the annealing process, the bottle decreases in volume due to shrinkage. The analysis of pressure resistance shows that the cracking always starts around the bottle label, i.e., around the cylindrical shape. The smaller is the cylinder, the greater is the pressure it can withstand before bursting and so annealing the bottle can increase the pressure resistance due to the bottle contraction that occurs. If the shape of the label area can be simplified to the shape of an ideal cylinder, with a much smaller wall thickness of the diameter of a given cylinder, then, in the label part of the bottle wall, there would occur the so-called membrane stress state. For this case, in an ideal cylindrical tank, the peripheral and longitudinal stresses are calculated by Formulas (1) and (2) respectively (these stresses are also the main stresses). Then, based on the Huber‒Mises‒Hencky hypothesis for the plane stress state, the reduced stress for known principal stresses can be calculated using Formula (3).

(1) $$\sigma_{1}=\frac{p\cdot R\left(p\right)}{g\left(p\right)}$$

(2) $$\sigma_{2}=\frac{p\cdot R\left(p\right)}{2\cdot g\left(p\right)}$$

(3) $$\sigma_{r}=\sqrt{\sigma_{1}{}^{2}+\sigma_{2}{}^{2}-\sigma_{1}\cdot \sigma_{2}}=\frac{\sqrt{3}}{2}\cdot \frac{p\cdot R\left(p\right)}{g\left(p\right)}$$

where $\sigma_{1}$ is the peripheral stresses in the wall of an ideal cylinder assuming a membrane state of stress; $\sigma_{2}$ is the longitudinal stresses in the wall of an ideal cylinder assuming a membrane state of stress; $\sigma_{r}$ is the reduced stress based on the Huber‒Mises‒Hencky hypothesis for a plane state of stress; p is the pressure of water enclosed inside the bottle; R(p) is the radius of an ideal cylinder that approximates the shape of the bottle label area, which is pressure-dependent and increases as the pressure increases; and g(p) is the wall thickness of the ring with the average radius of an ideal cylinder, which approximates the shape of the bottle label area, depends on the pressure, and decreases with increasing pressure.

Analysis of Formula (3) shows that the stress generated in the bottle wall material increases as a power function as the pressure of water enclosed in the bottle increases. The exponent is greater than one, because the pressure is also included as a function of the bottle radius (this radius increases with increasing pressure) and as a function of thickness (the thickness decreases with increasing pressure). In other words, the more the volume of the bottle increases (including the radius) during the pressure increase, the more the stress in the bottle wall increases, reaching the value of critical stress at which the bottle wall breaks. Thus, the value of the bursting pressure of the bottle is not an explicit measure correlating the phase structure of the bottle with the mechanical strength of the bottle material (which is determined by the “critical stress” at which the material decoheres, and this critical stress is due to the phase structure of the bottle), because pressure resistance is also affected by the geometry of the bottle wall (its diameter and thickness) and the rigidity of the bottle material, which is also affected by the phase structure. The greater is the rigidity of the bottle material (which is affected by the phase structure of the bottle), the smaller is the volume increase of the bottle as the pressure increases and therefore the smaller is the pressure increase in the bottle wall, i.e., the higher is the pressure of the water enclosed in the bottle for the wall pressure to reach the critical stress value at which the material breaks. It should be added that, for greater crystallinity, the material is more rigid, thus it decreases its volume less as the pressure increases. 

From the above analysis, it follows that the annealing process itself influenced the pressure resistance, but whether the annealing was free or in the bath did not have a statistically significant effect on the pressure resistance. On the other hand, annealing in the bath caused more shrinkage, which resulted in a smaller bottle diameter than free annealing, but the pressure resistance was comparable. This can be explained by the fact that, in annealing in the bath, the oriented amorphous phase had to disappear much more than in free annealing, which reduced the maximum stress (critical stress) that the bottle wall could withstand. In other words, reducing the diameter of the bottle due to shrinkage after the annealing process in the bath, which should increase the pressure resistance, was compensated by the loss of orientation of the amorphous phase (as a result of which, the maximum stress that the bottle wall could withstand was reduced). However, to confirm this explanation, more tests need to be carried out, especially regarding the microstructure examination of the material. In summary, because of the contraction occurring, there is no clear correlation between the amount of oriented amorphous phase and the pressure resistance. The analysis of the literature shows that, with the reduction of the oriented amorphous phase, the mechanical strength of the material decreases [3,4], but, according to Formula (3), with the decrease in volume (due to shrinkage), the mechanical strength increases. To determine such correlations, it would be necessary to study the relationship between the degree of orientation of the amorphous phase and the pressure resistance with the elimination of shrinkage. Thus, the analysis of the tests shows that it would be necessary to carry out pressure tests with a changed degree of orientation of the amorphous phase at a constant volume and at a changed volume, but with a constant degree of orientation of the amorphous phase. 

### 4.4. DOE Analysis of the Influence of the Blow Mold Temperature and Hot Fill Method on Pressure Resistance and Thermal Stability of the Bottle in a Two-Factor Plan—NoE: 17–28

**Figure 15:**
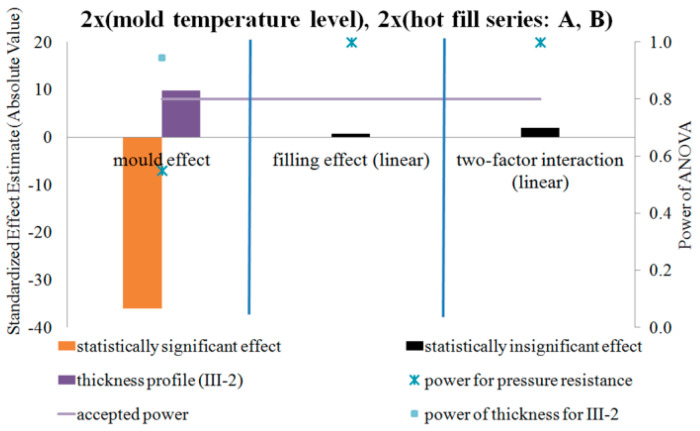
The results of testing the impact of the blow mold temperature and the hot filling method in the 2x2 plan on the pressure resistance of the bottle material for filling Methods A and B, together with the results of the analysis of the power of the tests carried out. Effects that do not meet the adopted assumption with a 5% probability of making the first type error are marked in black (NoE: 17–20).

**Figure 16:**
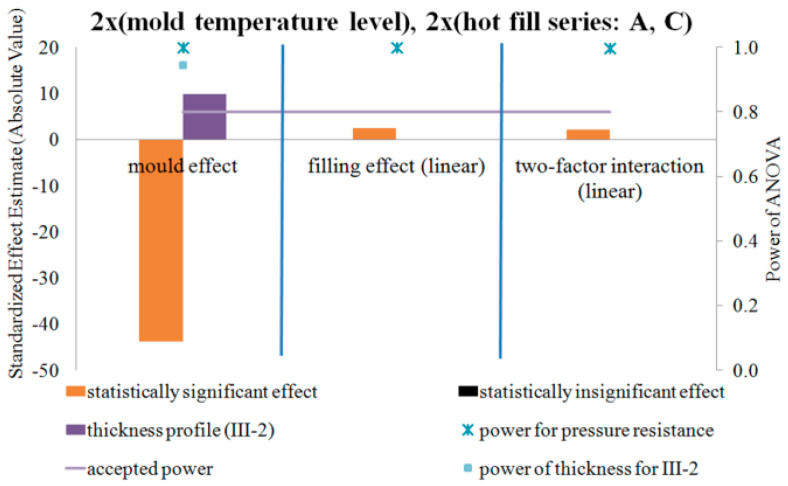
The results of testing the impact of the blow mold temperature and the hot filling method in the 2x2 plan on the pressure resistance of the bottle material for filling Methods A and C, together with the results of the analysis of the power of the tests carried out. Effects that do not meet the adopted assumption with a 5% probability of making the first type error are marked in black (NoE: 21–24).

**Figure 17:**
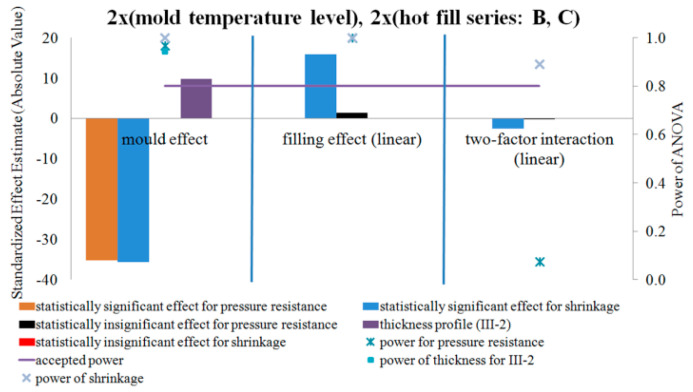
The results of testing the impact of the blow mold temperature and the hot filling method in the 2x2 plan on the pressure resistance, and thermal shrinkage of the bottle material for filling Methods B and C, together with the results of the analysis of the power of the tests carried out. The black and red colors indicate the effects that do not meet the adopted assumption of a 5% probability of making a first type error (NoE: 25–28).

Figure 15, Figure 16 and Figure 17 show the results of testing the impact of the blow mold temperature and hot filling method in a 2x2 plan on the pressure resistance and thermal shrinkage of the bottle material for hot filling Methods “A” and “B”, “A” and “C”, and “B” and “C”, respectively. The figures also show the results of the power analysis of the conducted analyses. For comparison, the figures also depict the effect of the blow mold temperature on the change in bottle thickness around Point III-2 of thickness measurement, i.e., around the start of bottle cracking during a pressure resistance test.

Figure 15, Figure 16 and Figure 17 show that the analysis of the 2x2 experiments (two-factor bivalent ANOVA) gives comparable results to those from the analysis of the 1x2 experiments (one-factor bivalent ANOVA—Figure 11, Figure 12, Figure 13 and Figure 14), although from the 2x2 plan it is more difficult to obtain statistically significant relationships between independent variables and a dependent variable. This is due to the fact that, in the 2x2 plan, it is not the arithmetic means of each series that are analyzed, but the arithmetic means of two series for each factor. It follows that the analysis of the 2x2 experiments is less sensitive to differences but allows easier interpretation of results as to the main effects as well as the determination and analysis of cross-effects between factors, which cannot be done for a single-factor plan. One-way plans can only be compared with the main effects of multi-factor plans.

Figure 15, Figure 16 and Figure 17 show that, regardless of whether a hot fill was not made or a hot fill was made and annealed, increasing the temperature of the blow mold strongly reduced the pressure resistance. Regardless of whether the mold was hot or cold, the annealing process after hot filling did not affect the pressure resistance. Increasing the temperature of the blow mold strongly reduced the thermal shrinkage of the bottle (increased thermal stability), while annealing in the bath resulted in a stronger thermal shrinkage. It follows that the hydrostatic pressure from the water stored in the bottle during free annealing statistically significantly counteracted the reduction in volume (shrinkage) during annealing. This effect could also be affected by the environment (air temperature) during free annealing, as a result of which the temperature of the bottle wall was cooler on the outer surface, which also counteracted the bottle’s shrinkage. In other words, to test the response of a bottle material to the process of annealing in hot water, this process should be tested by bath annealing.

Compared to the lack of annealing, bath annealing increased the pressure resistance of the bottle, but this may be due to a reduction in the diameter of the bottle and not a change in microstructure, as stated above. Further experimental studies are needed on representative samples cut from the surface of the bottles to eliminate the effect of changing the shape of the bottle during annealing by hot filling.

Figure 15 of the analysis plan of the study of the impact of the blow mold temperature and hot filling (lack of hot fill, Series “A”, as well as free annealing during hot fill, Series “B”) shows that there are no effects of interaction between the temperature of the blow mold and the hot fill on the change in pressure resistance. Figure 16 of the analysis plan of the study of the impact of the blow mold temperature and hot fill (lack of hot fill, Series “A”, as well as bath annealing during hot fill, Series “C”) shows that there are interaction effects between the temperature of the blow mold and the hot fill on the pressure resistance of the bottle. It follows that increasing the temperature of the blow mold reduces the effect of bath annealing, in comparison with the lack of annealing, on the pressure resistance (and vice versa, i.e., bath annealing instead of a lack of annealing reduces the effect of increasing the blow mold temperature on the pressure resistance). Figure 17 of the analysis plan of the study of the impact of the blow mold temperature and the annealing method during hot filling (Series “B” and “C”) shows that there are effects of the interaction between the temperature of the blow mold and the method of annealing during hot filling on the thermal shrinkage of the bottle. It follows that increasing the temperature of the blow mold reduces the effect of annealing in the bath relative to free annealing during hot filling on the thermal shrinkage of the bottle (and vice versa, i.e., annealing in the bath relative to free annealing reduces the effect of increasing the temperature of the blow mold on the thermal shrinkage of the bottle).

The problem of the occurrence of a high probability of errors of the second type (low power of statistical tests) should also be emphasized. Thus, from the results in Figure 15, Figure 16 and Figure 17, it appears that there is a very high probability of adopting the null hypothesis that independent variables do not affect dependent variables when it is statistically significant for analysis:

- the influence of the blow mold temperature on the bottle pressure resistance regardless of whether hot fill has occurred (Series “A”) or free annealing during hot filling has occurred (Series “B”); and
- the impact of the interaction between the temperature of the blow mold and the method of annealing during hot filling (free or in a bath) on the pressure resistance of the bottle.

Most likely, for the interaction effect, there is a statistically significant effect of independent variables on dependent variables, although to confirm the assumptions it would be necessary to repeat the test with a larger number of bottles in the measurement series.

## 5. Summary

Preliminary tests determining the sample size required to not exceed the maximum acceptable error indicate that 15 bottles should be produced in each measurement series. For measuring the weight of an empty bottle, weight of bottle filled with water, bottle pressure resistance, measurements required for calculating the blow kinetics coefficients, and thickness measurement statistical analysis showed that, in order for the tests to be reliable, various numbers of bottles and measurement repetitions for one bottle should be performed. This is an important novelty presented in the article. However, it should be very strongly emphasized that, in terms of the acceptable test power level of 80%, even the entire 15-bottle measurement series is too small a sample size for measurement of the thickness and dimensions needed to calculate the blow kinetics coefficients.

When measuring the density of the material by the gradient column method and the degree of crystallinity by the DSC method, the sample size that is required to not exceed the maximum acceptable error is too high, which exceeds the financial possibilities. In this case, the sample size was determined by the minimum sample size with which it is possible to carry out statistical analysis, and it was assumed that, from the population of 15 bottles produced for each of Series “A”, “B”, and “C”, three bottles would be drawn for which degree of crystallinity and density tests would be carried out with a single measurement repetition. This sample size was sufficient, in terms of the acceptable test power level of 80%, for the density test, but for the DSC crystallinity test the assumed sample size was definitely too small.

The tests of the bottle material density, material crystallite, and relaxation of amorphous phase, relative to the preform material density, material crystallite, and relaxation of amorphous phase, show that microcavity effects occur during the deformation of the material, and that these are associated with the orientation of the microstructure.

The kinetics of blow molding is puzzling and shows that there is a gradient of flow of the bottle material over the thickness of the bottle wall during blowing. Regardless of the temperature of the blow mold, the inner wall of the bottle was more circumferentially deformed than the outer wall of the blown bottle. Thus, as a result of orienting the macromolecules, it strengthened more strongly as a result of blowing than the outer wall. Thus, during the blow, it is “easier” to move from the outside of the bottle’s shoulders towards the label (and from around the label towards the bottom). However, a difference between a cold and hot mold occurs after the wall of the blown bottle comes into contact with the wall of the blow mold. 

## Figures and Tables

**Table 1:** Interpretation of the results of the Levene and post-hoc Scheffé statistical tests for the SBM process stabilization.

		Test of Homogeneity of Variances between Samples (Levene Test)	
		Homogeneity of Variance Preserved	Homogeneity of Variance Not Preserved (Welch Correction Should Be Introduced)
Test of difference between test averages (post-hoc Scheffé test)	The difference between bottle means and mean for whole series is insignificant for more than 90% of bottles in the series	The process is stabilized due to the properties of the bottle	The process is stabilized due to the properties of the bottles but there is an uncontrolled factor affecting the measured properties of the bottles
	The difference between bottle means and mean for whole series is significant for more than 10% of bottles in the series	The process is not stabilized due to the properties of the bottle	The process is not stabilized due to the properties of the bottle, perhaps as a result of the influence of an uncontrolled factor affecting the measured properties of the bottle

**Table 2:** Plan for one-factor, bivalent experiment described in this article for testing the thickness profile and blow kinetics (NoE, Number of Experiment) (see Figures 2–4).

NoE	Factor—SBM Process (Blow Mold Temperature)	
1	−1 (cold mold)	1 (hot mold)

**Table 3:** Plans for six one-factor, bivalent experiments described in this article for testing the degree of crystallinity, density, and relaxation of the amorphous phase (NoE, Number of Experiment) (see Figures 5–8).

NoE	Factor—SBM Process with Hot Fill Process		
	First Value (−1)	Second Value (1)	
		SBM Process (blow Mold Temperature)	Hot Fill Process
2	preform	cold	A—lack
3	preform	cold	B—free
4	preform	cold	C—bath
5	preform	hot	A—lack
6	preform	hot	B—free
7	preform	hot	C—bath

**Table 4:** Plans for nine one-factor, bivalent experiments described in this article for testing the pressure resistance and thermal stability (NoE, Number of Experiment) (see Figures 11–14).

NoE	Factor—SBM Process (Blow Mold Temperature)		Factor—Hot Fill Process	
8	−1(cold mold)	1 (hot mold)	A	
9	−1(cold mold)	1 (hot mold)	B	
10	−1(cold mold)	1 (hot mold)	C	
11	cold mold		−1(A)	1(B)
12	hot mold		−1(A)	1(B)
13	cold mold		−1(A)	1(C)
14	hot mold		−1(A)	1(C)
15	cold mold		−1(B)	1(C)
16	hot mold		−1(B)	1(C)

**Table 5:** Plans for three two-factor, bivalent experiments described in this article for testing pressure resistance and thermal stability (NoE, Number of Experiment) (see Figures 15–17).

Hot Fill Combination A–B	Main Factors		Interaction Factor	Hot Fill Combination A–C	Main Factors		Interaction Factor	Hot Fill Combination B–C	Main Factors		Interaction Factor
NoE	Mold	Hot Fill		NoE	Mold	Hot Fill		NoE	Mold	Hot Fill	
17	−1(cold)	−1(A)	1	21	−1(cold)	−1(A)	1	25	−1(cold)	−1(B)	1
18	−1(cold)	1(B)	−1	22	−1(cold)	1(C)	−1	26	−1(cold)	1(C)	−1
19	1(hot)	−1(A)	−1	23	1(hot)	−1(A)	−1	27	1(hot)	−1(B)	−1
20	1(hot)	1(B)	1	24	1(hot)	1(C)	1	28	1(hot)	1(C)	1

## Appendix A

**Table A1:** Results of the thickness sample size for each measuring point and for the dimensions of each blow kinetics mark of each bottle in the measurement series.

Measurement			Acceptable Maximum Error for One Bottle, ${∆}_{max}\;(\mathrm{mm})$	Estimated Population Standard Deviation for One Bottle, $\sigma_{p}\;(\mathrm{mm})$	Sample Size for One Bottle, n_1_ and n_2_	
					Minimal	Accepted
Thickness profile measurement (n₁)	I	1	0.01	0.0110	5	5
		2	0.01	0.0060	2	5
		3	0.01	0.0057	2	5
	II	1	0.01	0.0049	1	3
		2	0.01	0.0036	1	3
		3	0.01	0.0014	1	3
	III	1	0.01	0.0081	3	3
		2	0.01	0.0029	1	3
		3	0.01	0.0021	1	3
	IV	1	0.01	0.0025	1	3
		2	0.01	0.0025	1	3
		3	0.01	0.0017	1	3
	V	1	0.01	0.0078	3	5
		2	0.01	0.0091	4	5
		3	0.01	0.0078	3	5
Measurement of the dimensions needed to calculate the blow kinetics coefficients (n₂)	I	a	1	0.6645	2	5
		p1	1	0.5957	2	5
		p2	1	0.3895	1	5
		p3	1	0.7818	3	5
		e	1	0.6001	2	5
		f	1	0.8177	3	5
	II	a	1	0.9441	4	5
		p1	1	0.9702	4	5
		p2	1	1.1140	5	5
		p3	1	1.0187	4	5
		e	1	0.5242	2	5
		f	1	0.3668	1	5
	III	a	1	0.6626	2	5
		p1	1	1.0666	5	5
		p2	1	0.9207	4	5
		p3	1	1.1038	5	5
		e	1	0.5798	2	5
		f	1	0.7897	3	5
	IV	a	1	1.0952	5	5
		p1	1	1.0532	5	5
		p2	1	1.1179	5	5
		p3	1	0.9625	4	5
		e	1	0.5009	1	5
		f	1	0.6327	2	5
	V	a	- no measurements were taken due to the fact that the mark on the bottle practically did not change its position and dimensions relative to the mark on the preform			
		p1				
		p2				
		p3				
		e				
		f				

**Table A2:** The results of the sample size for the measurement series (number of bottles in the measurement series) of thickness measurement for each measurement point (n₃), and the dimensions of each blow kinetics determination (n₄).

Measurement			Acceptable Maximum Measurement Error for the Entire Measurement Series, ${∆}_{max}\;(\mathrm{mm})$	Estimated Standard Deviation in the Population for the Whole Measurement Series (Standard Deviation between Means for all 30 Bottles of Hot Mold, after Eliminating Statistically Different Values by Scheffé Tests—Table A5), $\sigma_{p}$ **(mm)**	Sample Size for the Measurement Series (Number of Bottles in the Series), **n_3_ and n_4_**	
					Minimal	Accepted
Thickness profile measurement (n₃)	I	1	0.01	0.0196	15	15
		2	0.01	0.0105	5	15
		3	0.01	0.0117	6	15
	II	1	0.01	0.0045	1	3
		2	0.01	0.0047	1	3
		3	0.01	0.0050	1	3
	III	1	0.01	0.0030	1	3
		2	0.01	0.0034	1	3
		3	0.01	0.0042	1	3
	IV	1	0.01	0.0045	1	3
		2	0.01	0.0039	1	3
		3	0.01	0.0046	1	3
	V	1	0.01	0.0136	8	12
		2	0.01	0.0113	5	12
		3	0.01	0.0172	12	12
Measurement of the dimensions needed to calculate the blow kinetics coefficients (n₄)	I	a	1	0.7641	3	5
		p1	1	0.8670	3	5
		p2	1	0.6190	2	5
		p3	1	0.8278	3	5
		e	1	0.8101	3	5
		f	1	0.9646	4	5
	II	a	1	1.1002	5	8
		p1	1	1.0002	4	8
		p2	1	1.1033	5	8
		p3	1	1.3454	7	8
		e	1	0.6670	2	8
		f	1	0.6007	2	8
	III	a	1	1.0500	5	10
		p1	1	1.4741	9	10
		p2	1	1.2206	6	10
		p3	1	1.4558	9	10
		e	1	0.6974	2	10
		f	1	0.8004	3	10
	IV	a	1	1.2130	6	10
		p1	1	1.4508	9	10
		p2	1	1.3856	8	10
		p3	1	1.4256	8	10
		e	1	0.7516	3	10
		f	1	1.1542	6	10
	V	a	- no measurements were taken due to the fact that the mark on the bottle practically did not change its position and dimensions relative to the mark on the preform			
		p1				
		p2				
		p3				
		e				
		f				

**Table A3:** The results of the sample size for the measuring Series “A”, “B”, and “C” (number of bottles in the measuring series) for measuring the burst pressure of the bottle (n_11_) and the weight of the bottle filled with water (n_12_).

Measurement		Acceptable Maximum Measurement Error for the Entire Measurement Series, ${∆}_{max}$	Estimated Standard Deviation in the Population for the Whole Measurement Series (Standard Deviation between Means for all 30 Bottles of Hot Mold, after Eliminating Statistically Different Values by Scheffé Tests—Table A5, $\sigma_{p}$	Sample Size for the Measurement Series (Number of Bottles in the Series), n₁₁ and n₁₂		
				Minimal	Accepted for Series “A”, “B”, and “C”	Accepted for the Whole Study
Pressure resistance measurement (n_5_, n_6_, n_7_, n_11_)	Series “A”	0.2 bar	0.3417 bar	12	n_5_ = 15	n_11_ = 15
	Series “B”	0.2 bar	0.3258 bar	11	n_6_ = 15	
	Series “C”	0.2 bar	0.3199 bar	10	n_7_ = 15	
Bottle weight measurement (n_8_, n_9_, n_10_, n_12_)	empty bottle (Series “A”, “B”, and “C”)	1 g	0.0353 g	1	3	n_12_ = 15
	filled bottle for Series “A”	1 g	0.4758 g	1	n_8_ = 3	
	filled bottle for Series “B”	5 g	8.5403 g	12	n_9_ = 15	
	filled bottle for Series “C”	5 g	9.145 g	13	n_10_ = 15	

**Table A4:** The results of the sample size for the measuring Series “A”, “B”, and “C” (number of bottles in the measuring series) for measuring the degree of crystallinity by the DSC method and the density of the material in a gradient column.

Measurement		Acceptable Maximum Measurement Error for the Entire Measurement Series, ${∆}_{max}$	Estimated Standard Deviation in the Population (Standard Deviation of Measurements for Three Randomly Selected Bottles out of the 30 Bottles), $\sigma_{p}$	Measurement Sample Size (Number of Bottles in the Series), n	
				Minimal	Adopted for Series “A”, “B”, and “C”
Measurement of crystallinity by DSC method	Series “A”	1%	1.94%	15	(3)
	Series “B”	1%	1.83%	13	(3)
	Series “C”	1%	0.97%	4	(3)
Density measurement in a gradient column method	Series “A”	0.001 g/cm^3^	0.0006 g/cm^3^	2	3
	Series “B”	0.001 g/cm^3^	0.0012 g/cm^3^	6	(3)
	Series “C”	0.001 g/cm^3^	0.0013 g/cm^3^	7	(3)

**Table A5:** Levene and post-hoc Scheffé test results for thickness measurement at measurement points (with a repeat of three and five times the measurement according to Table A1) and dimensions needed to calculate the blow kinetics coefficients at measurement points (with a repeat of five times the measurement according to Table A1) for 30 bottles manufactured in the Series “A” of the SBM process with a hot mold and for 15 bottles produced in the Series “A” of the SBM process with a cold mold.

Variable		Cold Mold (15 Bottles in Series “A”)			Hot Mold (30 Bottles in Series “A”)		
		Levene Test of Homogeneity of Variances	Number of Bottles Significantly Different from the Average Value of Whole Series—(Bottle Number)	Percent of Outliers—Stabilization of the SBM Process	Levene Test of Homogeneity of Variances	Number of Bottles Significantly Different from the Average Value of Whole Series—(Bottle Number)	Percent of Outliers—Stabilization of the SBM Process
Thickness profile	I-1	0.005—NO	1—(3)	7%—YES	0.000—NO	1—(13)	3%—YES
	I-2	0.000—NO	1—(15)	7%—YES	0.000—NO	0	0%—YES
	I-3	0.000—NO	0	0%—YES	0.000—NO	0	0%—YES
	II-1	0.000—NO	1—(3)	7%—YES	0.000—NO	0	0%—YES
	II-2	0.000—NO	1—(13)	7%—YES	0.000—NO	0	0%—YES
	II-3	0.000—NO	1—(13)	7%—YES	0.000—NO	0	0%—YES
	III-1	0.000—NO	0	0%—YES	0.000—NO	0	0%—YES
	III-2	0.000—NO	1—(10)	7%—YES	0.000—NO	0	0%—YES
	III-3	0.000—NO	0	0%—YES	0.000—NO	0	0%—YES
	IV-1	0.000—NO	1—(3)	7%—YES	0.000—NO	1—(4)	3%—YES
	IV-2	0.000—NO	0	0%—YES	0.000—NO	0	0%—YES
	IV-3	0.000—NO	1—(13)	7%—YES	0.000—NO	0	0%—YES
	V-1	0.002—NO	0	0%—YES	0.000—NO	0	0%—YES
	V-2	0.000—NO	0	0%—YES	0.000—NO	0	0%—YES
	V-3	0.000—NO	0	0%—YES	0.000—NO	0	0%—YES
Dimennsions of bottle kinetics markings	a-I	0.007—NO	0	0%—YES	0.000—NO	0	0%—YES
	a-II	0.004—NO	0	0%—YES	0.000—NO	0	0%—YES
	a-III	0.000—NO	1—(12)	7%—YES	0.003—NO	0	0%—YES
	a-IV	0.002—NO	1—(2)	7%—YES	0.000—NO	0	0%—YES
	p1-I	0.004—NO	0	0%—YES	0.000—NO	0	0%—YES
	p1-II	0.000—NO	1—(2)	7%—YES	0.002—NO	0	0%—YES
	p1-III	0.028—NO	1—(13)	7%—YES	0.000—NO	0	0%—YES
	p1-IV	0.000—NO	0	0%—YES	0.000—NO	0	0%—YES
	p2-I	0.017—NO	0	0%—YES	0.004—NO	0	0%—YES
	p2-II	0.000—NO	2—(2, 12)	13%—NO	0.010—NO	0	0%—YES
	p2-III	0.000—NO	1—(2)	7%—YES	0.000—NO	0	0%—YES
	p2-IV	0.004—NO	0	0%—YES	0.000—NO	0	0%—YES
	p3-I	0.019—NO	1—(12)	7%—YES	0.009—NO	0	0%—YES
	p3-II	0.000—NO	1—(11)	7%—YES	0.000—NO	0	0%—YES
	p3-III	0.095—YES	1—(10)	7%—YES	0.000—NO	0	0%—YES
	p3-IV	0.030—NO	1—(2)	7%—YES	0.000—NO	0	0%—YES
	e-I	0.011—NO	1—(13)	7%—YES	0.000—NO	0	0%—YES
	e-II	0.000—NO	0	0%—YES	0.000—NO	1—(22)	3%—YES
	e-III	0.000—NO	1—(13)	7%—YES	0.000—NO	0	0%—YES
	e-IV	0.001—NO	1—(13)	7%—YES	0.000—NO	0	0%—YES
	f-I	0.000—NO	1—(15)	7%—YES	0.000—NO	0	0%—YES
	f-II	0.709—YES	0	0%—YES	0.002—NO	0	0%—YES
	f-III	0.002—NO	1—(12)	7%—YES	0.000—NO	0	0%—YES
	f-IV	0.000—NO	2—(9, 12)	13%—NO	0.000—NO	1—(27)	3%—YES
